# What is your diagnosis?

**DOI:** 10.4103/2589-0557.75032

**Published:** 2010

**Authors:** Priyanka Singhal, Rahul Dixit, Shivani Patel, Y. S. Marfatia

**Affiliations:** Department of Skin and VD, Government Medical College and SSG Hospital, Vadodara, Gujarat, India

A 45-year-old uncircumcised married male presented with asymptomatic growth on the glans penis of 8 months duration. There were no complaints or past history suggestive of sexually transmitted diseases (STDs) or pre-existing skin lesion. There was no history of trauma, any other dermatoses, or systemic disease. There was no history of STD in the partner. The patient was initially diagnosed as a case of genital warts and was being treated with podophyllin and cryotherapy, but there was no improvement.

**Figure 1:**
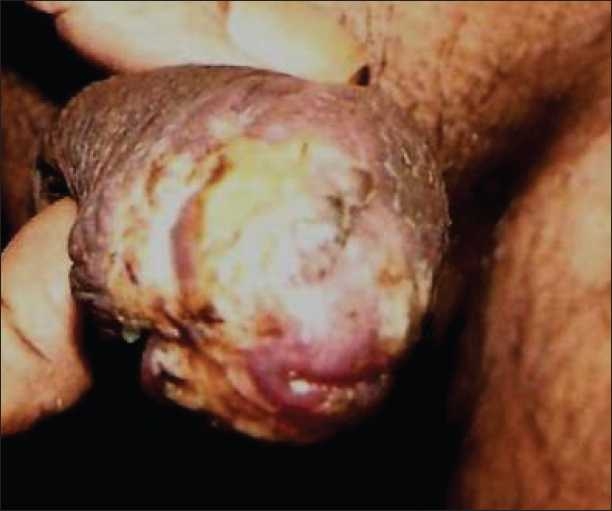
Hyperkeratotic, hypertrophic, verrucous plaque with mild crusting on glans penis

**Figure 2:**
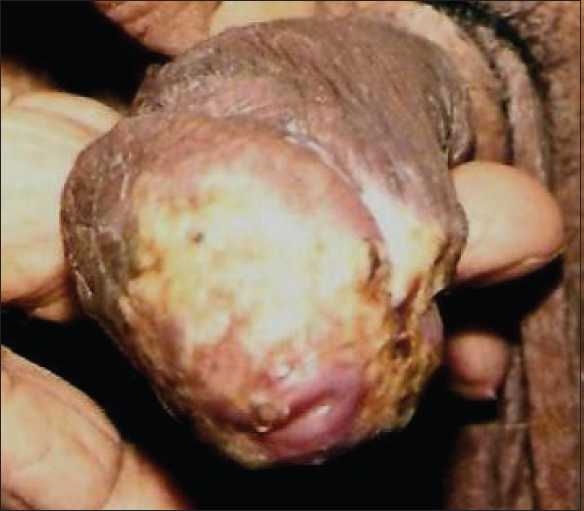
Hyperkeratotic plaque on glans penis

**Figure 3:**
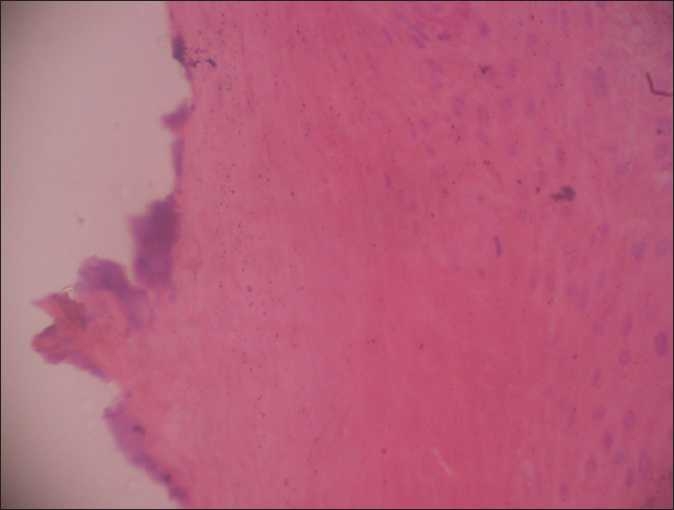
Massive thickening of stratum corneum with ortho- and parakeratotic hyperkeratosis (×10)

**Figure 4:**
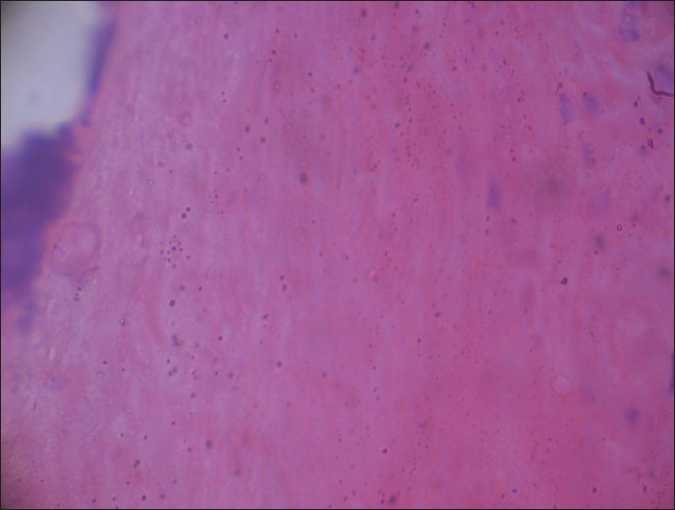
Massive thickening of stratum corneum with ortho- and parakeratotic hyperkeratosis (×40)

On examination, there was hyperkeratotic, hypertrophic, verrucous plaque with mild crusting on glans [Figures 1 and 2]. There was no inguinal lymphadenopathy. S. VDRL and S. HIV were nonreactive. Differentials considered were pseudoepitheliomatous keratotic and micaceous balanitis) and squamous cell carcinoma. Histopathologic examination showed massive thickening of stratum corneum with ortho- and parakeratotic hyperkeratosis. There was no evidence of malignant or premalignant changes at the base of the lesion [Figures 3 and 4].

**What is your diagnosis?**

Answer: Cutaneous Horn

Cutaneous horn (Cornu cutaneum) refers to a well-defined cone-shaped lesion with hyperkeratotic features. Approximately 30% of cutaneous horns are found on the face and scalp. Other common locations include sun-exposed areas, such as the ear, lip, chest, neck, and shoulder. They may, however, develop on areas not exposed to sunlight, such as the penis, mucosal lower lip, and nasal vestibule.[1,2]

Over 60% of lesions are benign; however, malignant and premalignant lesions might be associated with it. These conditions include actinic keratosis, squamous cell carcinoma, seborrheic keratosis, molluscum contagiosum, verruca vulgaris, trichilemmoma, Bowen’s disease, and basal cell carcinoma.

They are asymptomatic with variable size and form, such as cylindrical, conical, pointed, corrugated transversely and longitudinally, or curved like a ram’s horn. They are solitary, growing slowly over years to decades if left alone. The base of the horn may be flat, nodular, or crateriform.[3] Surrounding inflammation and an infiltrated base are unusual, but they may indicate malignancy. Tenderness at the base also favors malignancy. Risk factors for underlying malignancy include advanced age, male sex, large base or height-to-base ratio, and presence on a sun-exposed location.

The etiology of penile horns is uncertain, although they are often found in association with warts, phimosis, naevi, and in areas afflicted by trauma if present. As reported by Lowe and McCullogh, the condition may be benign in 42%–56% of cases, premalignant in 22%–37%, or frankly malignant in 20%–22%.[4]

Treatment options include wide surgical excision with careful histologic examination to exclude a focus of malignancy. If malignancy is present in a penile cutaneous horn, the treatment involves partial penectomy with or without regional lymph node dissection. Therapy with carbon dioxide or Neodymium YAG laser is used for patients who refuse surgery. Our patient was advised for surgical excision and its histopathologic examination to rule out any foci of malignant change.
